# New insights into the role of EMT in tumor immune escape

**DOI:** 10.1002/1878-0261.12093

**Published:** 2017-06-27

**Authors:** Stéphane Terry, Pierre Savagner, Sandra Ortiz‐Cuaran, Linda Mahjoubi, Pierre Saintigny, Jean‐Paul Thiery, Salem Chouaib

**Affiliations:** ^1^ INSERM UMR 1186 Integrative Tumor Immunology and Genetic Oncology Gustave Roussy EPHE Fac. de médecine ‐ Univ. Paris‐Sud University Paris‐Saclay Villejuif France; ^2^ Institut de Recherche en Cancérologie de Montpellier France; ^3^ U1194 INSERM Montpellier France; ^4^ Université Montpellier France; ^5^ Institut du Cancer Montpellier France; ^6^ INSERM U1052 CNRS UMR 5286 Cancer Research Center of Lyon France; ^7^ Université de Lyon France; ^8^ Centre Léon Bérard Lyon France; ^9^ Faculté de Pharmacie de Lyon ISPB Université Lyon 1 France; ^10^ LabEx DEVweCAN Université de Lyon France; ^11^ CNRS UMR 7057 Matter and Complex Systems Paris France; ^12^ Department of Biochemistry Yong Loo Lin School of Medicine National University of Singapore Singapore

**Keywords:** antitumor immunity, EMT, immune escape, tumor microenvironment

## Abstract

Novel immunotherapy approaches have provided durable remission in a significant number of cancer patients with cancers previously considered rapidly lethal. Nonetheless, the high degree of nonresponders, and in some cases the emergence of resistance in patients who do initially respond, represents a significant challenge in the field of cancer immunotherapy. These issues prompt much more extensive studies to better understand how cancer cells escape immune surveillance and resist immune attacks. Here, we review the current knowledge of how cellular heterogeneity and plasticity could be involved in shaping the tumor microenvironment (TME) and in controlling antitumor immunity. Indeed, recent findings have led to increased interest in the mechanisms by which cancer cells undergoing epithelial‐mesenchymal transition (EMT), or oscillating within the EMT spectrum, might contribute to immune escape through multiple routes. This includes shaping of the TME and decreased susceptibility to immune effector cells. Although much remains to be learned on the mechanisms at play, cancer cell clones with mesenchymal features emerging from the TME seem to be primed to face immune attacks by specialized killer cells of the immune system, the natural killer cells, and the cytotoxic T lymphocytes. Recent studies investigating patient tumors have suggested EMT as a candidate predictive marker to be explored for immunotherapy outcome. Promising data also exist on the potential utility of targeting these cancer cell populations to at least partly overcome such resistance. Research is now underway which may lead to considerable progress in optimization of treatments.

**Abbreviations**
APCs: antigen‐presenting cells
CAFs: cancer‐associated fibroblasts
CCL: C‐C motif chemokine ligand
CCR: C‐C motif chemokine receptor
COX2: cyclooxygenase‐2
cSMAC: central supramolecular activation cluster
CTC: circulating tumor cell
CTLA‐4: cytotoxic T lymphocyte‐associated protein 4
CTL: cytotoxic T lymphocyte
CXCL: C‐X‐C motif chemokine ligand
DC: dendritic cells
EMT: epithelial‐mesenchymal transition
EMT‐TF: epithelial‐mesenchymal transition transcription factor
Epi: epithelial
Erbb2: Erb‐b2 receptor tyrosine kinase 2
ESRP1: epithelial splicing regulatory protein 1
FFPE: formalin‐fixed paraffin‐embedded
FOXC2: forkhead box C2
FOXP3: forkhead box P3
GRHL2: grainyhead‐like transcription factor 2
GZM: granzyme
HIF‐1α: hypoxia‐induced factor 1 alpha
HNSCC: head and neck squamous cell carcinoma
ICAM: intercellular adhesion molecule‐1
ICOS: inducible T‐cell costimulator
IDO: indoleamine‐2,3‐dioxygenase
IFN: interferon
IL: interleukin
KLF8: kruppel‐like factor 8
LAG3: lymphocyte activating 3
LFA‐1: lymphocyte function‐associated antigen‐1
MDSC: myeloid‐derived suppressor cells
Mes: mesenchymal
MET: mesenchymal‐epithelial transition
MHC: major histocompatibility complex
MIC: MHC class I chain‐related protein
miRs: microRNA
MSI: microsatellite instability
NK: natural killer
NSCLC: non‐small‐cell lung cancers
PD‐1: programmed cell death protein‐1
PD‐L1: programmed cell death 1 ligand 1
PGE: prostaglandin E
PI3K: phosphatidyl inositol‐3‐kinase
Prf: perforin
PRRX1: paired‐related homeobox 1
pSMAC: peripheral supramolecular activation cluster
ROS: reactive oxygen species
RTK: receptor tyrosine kinase
SNAIL1: snail family zinc finger 1
TCR: T‐cell receptor
TGF‐β: tumor growth factor beta
TIL: tumor‐infiltrating lymphocyte
TIM3: T‐cell immunoglobulin mucin 3
TME: tumor microenvironment
TNC: tenascin‐C
TNF: tumor necrosis factor
Treg: regulatory T cell
TSP: thrombospondin
TWIST1: Twist family bHLH transcription factor 1
VEGF: vascular endothelial growth factor
ZEB: zinc finger E‐box binding homeobox

## 1 Introduction

Here, in a first part, we consider the role of the tumor microenvironment (TME) in the field of Immuno‐oncology, and then, introduce key notions regarding the epithelial‐mesenchymal transition (EMT), cell plasticity, resistance to chemotherapy and radiotherapy. In a second part, we review the current state of knowledge regarding the multifaceted manner by which EMT, and cancer cells with mesenchymal features, could influence the TME and modulate antitumor immunity. Finally, we discuss the potential impact and the need to integrate these discoveries to develop effective therapeutic strategies, as well as for the development of biomarkers of response for immunotherapies.

## 2 Tumor microenvironment and antitumor immunity

Although immune checkpoint blockers (anti‐PD‐1, anti‐PD‐L1, anti‐CTLA‐4) on T cells result in significantly improved survival in various metastatic cancer types [non‐small‐cell lung cancers (NSCLC), melanoma, renal carcinoma, bladder carcinoma, head and neck squamous cell carcinoma (HNSCC), and lymphoma] (Ansell, 2017; Borghaei *et al*., 2015; Brahmer *et al*., 2015; Burstein *et al*., 2017; Ferris *et al*., 2016; Garon *et al*., 2015; Hamid *et al*., 2013; Motzer *et al*., 2015; Reck *et al*., 2016; Rosenberg *et al*., 2016; Sharma *et al*., 2017b; Younes *et al*., 2016), a high fraction of patients with cancer fail to respond to these therapeutic interventions. This is manifested through different forms including intrinsic resistance, or through acquired resistance in patients initially responding (Restifo *et al*., 2016; Sharma *et al*., 2017a). Moreover, in the current setting, a number of cancer types respond poorly, such as prostate, breast, non‐microsatellite instability (non‐MSI) colon, and pancreatic cancers. While the exact reasons for this lack of response are largely unknown, it is interesting to note that even in good responders, the responses can significantly differ between cancer lesions in a given patient (O'Donnell *et al*., 2017). This underlies the importance of the TME and cancer niches establishing during cancer initiation and progression. Indeed, a developing tumor, to sustain its growth, must establish an immunosuppressive TME to neutralize the activation of immune responses (Gajewski *et al*., 2006; Joyce and Fearon, 2015; Kerkar and Restifo, 2012; Mantovani *et al*., 2008; Vasievich and Huang, 2011). All immune cell types [including dendritic cells (DC), natural killer (NK) cells, macrophages, neutrophils, B and T lymphocytes (CD4^+^ T helper 1 (T_H_1) and 2 (T_H_2), CD8^+^ cytotoxic T cells (CTL), memory cells, and regulatory T (Treg) cells)] may be present within or at the edge of a given tumor, or in a form of tertiary lymphoid structures located in the stromal compartment that gather multiple immune components similar to that found in secondary lymphoid organs (Fridman *et al*., 2012). The immune infiltrates may vary strikingly during stages of tumor development, from one patient to another, between different cancers, or cancer histotypes. Analysis of the ‘immune contexture’ in patient tumors revealed that the TME comprises a mosaic of immunosuppressive cells such as myeloid‐derived suppressor cells (MDSC) (Kumar *et al*., 2016; Marvel and Gabrilovich, 2015), cancer‐associated fibroblasts (CAFs) (Kraman *et al*., 2010; Salmon *et al*., 2012), tumor‐associated macrophages (Allavena and Mantovani, 2012; Ruffell *et al*., 2012), and Treg cells (Ghiringhelli *et al*., 2005; Whiteside, 2008). Immunoregulatory enzymes (arginase, COX2, INOS) and immunosuppressive substances produced by these cells such as IL‐10, tumor growth factor beta (TGF‐β), vascular endothelial growth factor, PGE2, or PD‐L1 can impede both the innate and adaptive immunities by inhibiting NK cells, CD4^+^ and CD8^+^ effector T cells, or by inhibiting DC maturation, through reducing major histocompatibility complex (MHC) molecules on the latter and costimulatory signals essential to activate naïve T cells. Together with this growing knowledge, a new hope has emerged that efficient targeting of the components of the TME could affect cancer in all of its stages.

Besides these different cell types, hypoxia is an essential metabolic element of the TME that shapes cellular plasticity and tumor heterogeneity (Keith and Simon, 2007; Pouyssegur *et al*., 2006). During tumor development, while cells residing close to blood vessels are relatively well oxygenated, those at more distant sites often face hypoxic stress, coinciding with O_2_ deprivation. Hypoxia conditioning leads to stabilization of HIF‐α proteins in these cells which mediate cellular adaptation to this stress by inducing expression of target genes, associated with changes in cell metabolism, behavior, and phenotype (Keith and Simon, 2007; Majmundar *et al*., 2010; Pouyssegur *et al*., 2006). The intensity and the duration of stress vary among the different cell types. Considerable evidence now suggests that hypoxia also impairs antitumor immunity through different mechanisms involving cancer cell plasticity or education of nonimmune cells toward an immunosuppressive phenotype (Corzo *et al*., 2010; Kumar and Gabrilovich, 2014; Noman *et al*., 2015).

The concept of immunoediting, originally embodied by the notion of tumor immunosurveillance, can recapitulate many of the salient features observed in the natural history of a cancer. It highlights the important interactions between the immune system and the tumor at various stages of development (Dunn *et al*., 2002; Mittal *et al*., 2014). In the first phase, ‘elimination’, a substantial number malignant cells are recognized by competent immune effector killer cells such as CTLs and NK cells through the activation of innate and adaptive immune routes. In a second phase, sporadic cancer cells managing to survive the immune attacks enter an ‘equilibrium’ phase where editing of the tumor occurs. The establishment of an immunosuppressive TME probably begins during this phase and will be further accentuated in final phase of the immunoediting process, called the ‘escape’ phase. This phase is characterized by malignant clones shaping the TME under a severe selection pressure and adaptation, and developing intrinsic mechanisms to escape immune detection and destruction (Zitvogel *et al*., 2006). This gives rise to clinically significant immunologically edited tumors. More research is required to decrypt the complex network of interactions between tumor, immune and nonimmune cells in the clinical setting (Galon *et al*., 2013). It is unlikely that immunoediting completely eradicates the cancer cell clones with highly immunogenic epitopes, as evidenced by notable responses observed in some patients under anti‐CTLA4 and anti‐PD1 therapies. Additionally, it is still unclear in this context why most patient tumors maintain high cancer cell heterogeneity and how this impacts on TME changes (and vice versa) as well as in mounting immune resistance (Holzel *et al*., 2013).

Over the past decade, EMTs, and transitional states between epithelial (Epi) and mesenchymal (Mes) states, have been suggested as critical mediators in metastatic progression, and therapy resistance, including chemo‐, radio‐, and targeted therapy resistances. In light of the current knowledge, cancer cells undergoing EMT, or harboring Mes characteristics, may also have profound influence on the cellular components present in the TME, including immune cells. Evidence is now accumulating that such cross‐talks might be key determinants in facilitating immune escape by tumors, with the potential to regulate immunotherapy efficacy.

## 3 EMT, cell plasticity, and cancer: basic principles

Early embryonic developmental stages are characterized by extensive plasticity in cellular organizations. Following cellularization or cleavages, cells expand their intercellular contact to progressively form a polarized layer called an epithelium. The cells within these sheets develop extensive apico‐basal cell surface polarity with actin microfilaments that are mostly concentrated at the cortex of the basolateral membranes, and with microtubules oriented baso‐apically that position cytoplasmic organelles, such as the Golgi, above the nucleus along the baso–apical axis. In polarized Epi cells, junctional complexes are localized on the lateral domain, and the basal domain interacts exclusively with the extracellular matrix forming a basal lamina through integrin receptors. These distinct junctional complexes, particularly E‐cadherin‐associated adherens junctions, contribute to maintaining apico‐basal polarity (Huang *et al*., 2012). Mes cells, on the contrary, are formed during gastrulation through EMT, a fundamental mechanism of development in most multicellular organisms (Lim and Thiery, 2012; Nieto *et al*., 2016). During gastrulation in higher vertebrates, Mes mesodermal and endodermal cells dissociate from an Epi cell layer called the epiblast and ingress through the primitive streak. Mes cells lose their apico‐basal polarity, redistribute their actin cytoskeleton throughout the cytoplasm, and remain connected to the basal surface through focal adhesions and intercellular punctate adhesions. These features contribute to the development of a front‐rear polarity during the acquisition of migratory behavior. Most of the ingressed cells then engage in the reverse process named mesenchymal‐epithelial transition (MET) to establish paraxial and lateral mesodermal structures. Organogenesis subsequently involves cycles of EMT and MET, such as seen in heart development. These successive rounds of EMT and MET in development are driven by multiple signaling pathways, including canonical Wnt, TGF‐β, and RTK pathways (Lim and Thiery, 2012). One of the most striking examples of EMT is the formation and subsequent extensive migrations of cells of the neural crest, a transient, embryonic structure in the dorsal neuroepithelium. These properties are proposed to have been hijacked by cancer cells to initiate invasion and metastasis (Thiery, 2002). Our current understanding of the mechanisms driving EMT in carcinoma has predominantly come from *in vitro* studies using a limited number of carcinoma cell lines. EMT is classically driven by transcriptional repressors commonly referred to as EMT transcription factors (EMT‐TF) including SNAIL1/2 and ZEB1/2, which directly repress E‐cadherin expression by binding to E‐boxes on its proximal promoter. TWIST and several other transcription factors (FOXC2, E47 (TFC3), KLF8, and PRRX1) also induce EMT. Although it is still unclear whether these factors directly regulate E‐cadherin expression (De Craene and Berx, 2013), they have multiple other target genes and may function downstream in canonical RTK, TGF‐β, and Wnt receptor signaling, among others (Lamouille *et al*., 2014). The miRs (microRNA) are also critically involved in maintaining the Epi state, with ZEB1 and miR‐200 family members forming negatively regulated feed‐back loops (Zhang and Ma, 2012). In addition to transcriptional and miR regulation, splicing mechanisms, post‐translational modifications, and epigenetic changes also significantly contribute to the EMT phenotype (De Craene and Berx, 2013). Epigenetics ensures a more stable position in the EMT spectrum as compared with the continuous expression of transcriptional repressors. For instance, DNA methylation and histone repressive marks are associated with a more Mes phenotype, whereas poised chromatin allows for a more plastic phenotype, residing in the intermediate EMT stages (Tam and Weinberg, 2013). Some transcription factors, such as GRHL2, originally shown to sustain E‐cadherin gene expression through its binding to its second intron (Cieply *et al*., 2012), may also participate in maintaining the stability of Epi state. GRHL2 thus protects carcinoma cells from transitioning from the intermediate Epi state to the Mes states. However, the forced expression of GRHL2 in Mes ovarian carcinoma cells cannot revert the cells to an Epi state (Chung *et al*., 2016). Evidence suggests that this irreversibility is primarily caused by heterochromatization of GRHL2 target genes. In recent years, studies have emphasized that Mes carcinoma cells have acquired stem cell properties (Chaffer *et al*., 2011; Mani *et al*., 2008) and a drug‐resistant phenotype (Gupta *et al*., 2009; Mitra *et al*., 2015; Singh and Settleman, 2010). However, there remain numerous questions regarding when and how carcinoma cells acquire a Mes phenotype, drug resistance, and stemness. For example, does it happen before or during dissemination through the lymph and blood vessels? Approximately 3% of luminal breast carcinoma cells express Mes markers and a significantly higher percentage is detected in triple‐negative breast tumors (Sarrio *et al*., 2008; Tan *et al*., 2014; Yu *et al*., 2013a). A longitudinal analysis of patients with metastatic breast cancer, who underwent several cycles of different targeted therapeutics to overcome refractoriness, finally became fully refractory once their tumors reached a Mes phenotype (Yu *et al*., 2013a). EMT in these carcinoma cells may be induced by the local microenvironment and hypoxia, priming certain cells for disseminating from the primary tumor. Stromal cells, including myofibroblast and inflammatory cells, could contribute as EMT inducers by secreting numerous growth factors and cytokines (Bai *et al*., 2015; Wyckoff *et al*., 2007). Overall, it appears that the intermediate Epi and Mes carcinoma phenotypes are more suitable for tumor progression and distant dissemination, thus making the EMT process a promising target for therapeutic intervention, to prevent or suppress these deleterious effects. A major issue is whether we can develop new therapeutic strategies based on the EMT concept (Antony *et al*., 2016; Chua *et al*., 2012). Moreover, the concept that EMT confers invasion, metastasis, stemness, and drug resistance need to be addressed in the clinical setting rather than with murine models. These studies will pave the way toward a deeper understanding of the most important properties conferred by EMT during dissemination.

## 4 EMT and resistance to chemotherapy and radiotherapy

EMT‐TFs have been found to mingle with stemness pathways and to induce resistance to chemotherapy and radiotherapy in various cancer models, *in vitro* and *in vivo* (Dave *et al*., 2012; Fischer *et al*., 2015; Sanchez‐Tillo *et al*., 2012; Zheng *et al*., 2015). The molecular pathways connecting chemotherapy/radiotherapy and EMT‐TFs strongly depend on the molecular type of the tumor (Deng *et al*., 2016; Tan *et al*., 2014). TGF‐β, NF‐kB, Wnt, FGF, and EGF/HER2 pathways, found to be activated in response to chemotherapy/radiotherapy, can stimulate EMT‐TF expression. Regulation by miRs is also to consider as certain miRs, found to be downregulated in resistant cells, directly suppress EMT‐TF expression, while increasing chemosensitivity in cells with a more Epi phenotype (Nantajit *et al*., 2015). A deregulation of the miR‐200 family/ZEBs axis appears to mediate docetaxel resistance of prostate cancer cells (Hanrahan *et al*., 2017; Puhr *et al*., 2012). miR‐21, a well‐known ‘oncomir’ (i.e., a miR with oncogenic properties), is associated with EMT, resistance to trastuzumab, as well as to chemotherapy (paclitaxel or doxorubicin) through the PTEN/AKT pathway in HER2‐positive breast cancer cells (De Mattos‐Arruda *et al*., 2015). A primary mechanism of chemoresistance involves drug elimination by increasing efflux of hydrophobic drugs regulated by ATP‐binding cassette (ABC) transporters, a family of energy‐dependent transporters including multidrug resistance protein 1 MDR1/P‐glycoprotein (ABCB1 gene) and multidrug resistance protein MRP (ABCC1 gene). EMT‐TFs including SNAIL and TWIST families bind to E‐boxes found in promoter regions of ABC genes (Saxena *et al*., 2011). Radiation and hypoxia also generate reactive oxygen species (ROS), and it was suggested that EMT programs can be activated by ROS elevation with potential consequences on drug or radiation resistance (Nantajit *et al*., 2015). The EMT‐TF SLUG has been found to antagonize p53‐mediated apoptosis by repressing PUMA, therefore promoting BCL2 function and cell survival (Kurrey *et al*., 2009). Radiotherapy and several chemotherapy agents affect genome integrity. Recent evidence shows that EMT‐TFs regulate genomic instability in various model systems by activating DNA damage response genes such as ERCC1, DNA ligase 1, and ATM (Deng *et al*., 2016; Hsu *et al*., 2010), or by regulating BRCA1 expression in breast carcinomas in the case of SLUG (Wu *et al*., 2012).

## 5 Cancer cell immune resistance

Aside from the creation of an immunosuppressive TME that one could consider as providing with tumor‐extrinsic mechanisms (van der Burg *et al*., 2016), immune escape can also be engaged by cancer cells themselves, as they face and trick the immune system. Identification and characterization of cancer cell‐intrinsic mechanisms have been the subject of intense research (van der Burg *et al*., 2016; Crespo *et al*., 2013; Dunn *et al*., 2002; Khong and Restifo, 2002; O'Donnell *et al*., 2017). For instance, cancer cells can escape the immune system of the host by hiding their tumor‐specific antigens. In particular, the immune cells are greedy for cancer cell expressing neoantigens, produced as a result of tumor‐specific ‘nonsynonymous’ mutations in gene‐coding regions, and cross‐presented by the antigen‐presenting cells (APCs) (Rooney *et al*., 2015). It was recently found that the tumor neoantigen spectrum strongly correlates with immunogenicity and with response to anti‐PD1 immunotherapy (McGranahan *et al*., 2016; Rizvi *et al*., 2015; Snyder *et al*., 2014). By losing some of their neoantigens, cancer cells thus manage to evade the immune system and immunotherapy treatments (Anagnostou *et al*., 2017). This can be achieved through different molecular events. Downregulation or loss of MHC class I proteins was observed a while ago and confirmed by high‐throughput studies (Garrido *et al*., 2010; Korkolopoulou *et al*., 1996; Rooney *et al*., 2015). MHC class I proteins associate with small peptide antigens (8–10 amino acids in length) to present them on the surface of cells and activate CD8^+^ T lymphocytes via the T‐cell receptor (TCR). Of note, TEIPP (T‐cell epitopes associated with impaired peptide processing) antigens may be presented by the residual MHC class I molecules of immune‐edited cancer cells, which could be exploited by vaccination approaches (van der Burg *et al*., 2016). Other defects in the antigen‐processing machinery involving beta‐2‐microglobulin (B2M), or transporter associated with antigen processing (TAP‐1 and TAP‐2), are also regarded as important mechanisms of immune escape (del Campo *et al*., 2014; Korkolopoulou *et al*., 1996). Likewise, alterations in the interferon‐gamma (IFN‐γ) signaling pathway are known to impact on antitumor responses (Gao *et al*., 2016). Cancer cells can also hinder the immune attacks by overexpressing antiapoptotic proteins such as BCL‐XL (Jazirehi *et al*., 2011).

## 6 The acquisition of a mesenchymal phenotype is associated with resistance to CTLs

**Figure 1:**
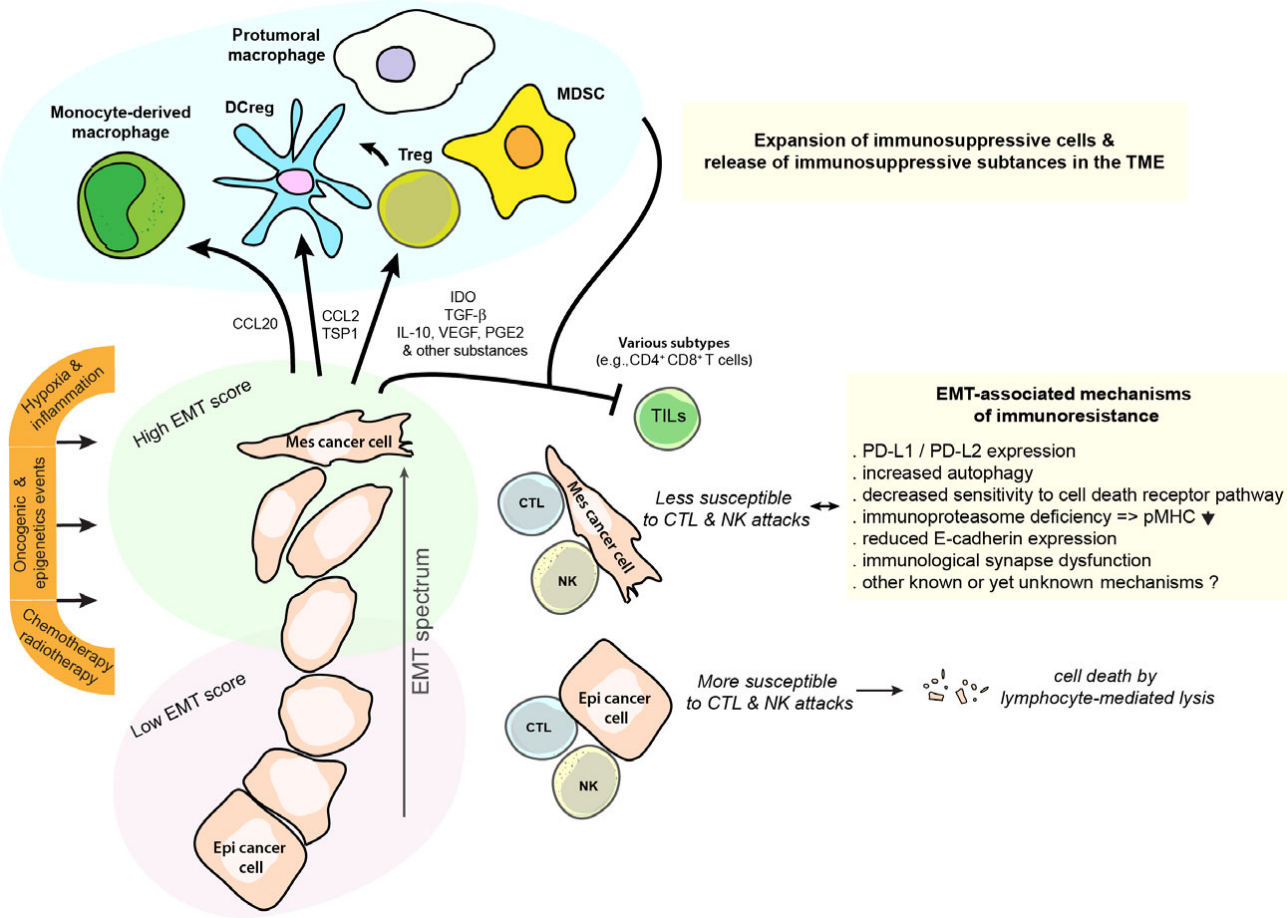
Cross‐talks between carcinoma cells, immunosuppressive cells, and immune effector cells within the tumor microenvironment in controlling antitumor immunity and immune escape.

The hypothesis that EMT might also contribute to immune escape of tumors was addressed by exploiting the human mammary carcinoma model MCF7 which underwent EMT, following stable expression of SNAIL or after prolonged exposure to tumor necrosis factor alpha (TNF‐α), exhibited reduced susceptibility to CTL‐mediated lysis (Akalay *et al*., 2013). MCF7 cells display pronounced Epi traits as assessed by their EMT scores when the EMTed MCF7 derivatives displayed various Mes states (Akalay *et al*., 2013). In this case, the protection from CTL‐mediated lysis appeared to be linked with the activation of an autophagic program, which may contribute to promote survival in these cells. A similar impairment of CTL‐mediated lysis was also noted in another model of MCF7 cells undergoing EMT (Akalay *et al*., 2015) wherein silencing of WISP2 (WNT1‐inducible signaling pathway protein 2) coincides with hyperactivity of TGF‐β signaling, EMT, and acquisition of stemness properties (Ferrand *et al*., 2014; Fritah *et al*., 2008). In this instance, the autophagy status did not appear to be involved in the resistant phenotype; however, a disruption of TGF‐β signaling in these cells by treatment with A83‐01, an inhibitor of TGF‐β‐related type I receptors ALK5, ALK4, and ALK7, effectively decreased resistance to CTLs. These findings suggest that key developmental pathways such as TGF‐β signaling in Mes cancer cells can mediate immune resistance to CTLs. This further suggests that different Mes cancer cell variants along the EMT spectrum could engage various mechanisms of resistance (Fig. 1). In this regard, we recently investigated the expression levels of PD‐L1 in the Mes MCF7 derivatives (Noman *et al*., 2017). While PD‐L1 was highly expressed in MCF7 cells silenced for WISP2, other cell lines had little or no expression of PD‐L1 similar to that found in the parental MCF7 cells. Additionally, it was demonstrated that PD‐L1 production by these MCF7‐shWISP2 cells participates in modulating immunoresistance toward CTLs. The EMT‐TF ZEB1 was identified as an important regulator of PD‐L1 expression in this system, supporting a rationale for EMT blockers as an alternative to control PD‐L1 expression and boost immunotherapeutic responses.

Tumor hypoxia is an important parameter to consider as a driver of EMT, tumor heterogeneity, and tumor immune escape. Using a model of lung adenocarcinoma (IGR‐Heu) cells derived from a nonmetastatic patient, we noted hypoxic stress‐induced phenotypic diversification along the EMT spectrum (Terry *et al*., 2017). We observed that the shift toward a more Mes phenotype in hypoxia‐exposed cells is only observed in a fraction of these NSCLC cells. In other words, while some cells undergo an EMT, others do not, or not to the same extent, therefore promoting cancer cell heterogeneity. Further analysis of cancer subclones with pronounced Epi or Mes phenotypes emerging from such hypoxic stress revealed that Mes subclones exhibited an increased propensity to resist CTL cell‐mediated lysis. The observed resistance to the autologous CTL Heu171 clone attack can be partially explained by the absence of detectable E‐cadherin in the Mes IGR‐Heu cancer variants, as the cytolytic function of CTL Heu171 relies on integrin CD103 (αΕβ7 integrin), and its interactions with its preferred ligand, E‐cadherin (Franciszkiewicz *et al*., 2013; Le Floc'h *et al*., 2007). Of note, CD103 is preferentially expressed by tumor‐infiltrating lymphocytes (TILs), and its expression in tumor tissue in NSCLC and ovarian cancers was associated with a good outcome (Djenidi *et al*., 2015; Webb *et al*., 2014). We also suspect the contribution of resistance mechanisms independent of the E‐cadherin–CD103 interaction, as inhibition of TGF‐β signaling minimized resistance to CTL‐mediated killing without apparent changes in expression of E‐cadherin in Mes cancer clones (Terry *et al*., 2017).

It is known that CTLs mainly use the perforin/granzyme pathway to destroy target cells (Barry and Bleackley, 2002). However, especially when this pathway is affected, the death may also be triggered through the activation of caspase‐dependent or caspase‐independent death receptor pathways with engagement of TNF‐related apoptosis‐inducing ligand (TRAIL) or FAS at the surface of cancer cells (Barry and Bleackley, 2002). Using the pancreatic PANC1 model, it has been demonstrated that cells with forced expression of Brachyury, an EMT inducer, had decreased susceptibility to lymphocyte‐mediated killing compared to control cells (Hamilton *et al*., 2014). By using experimental conditions in which target cancer cells were cocultured with effector CTLs for a relatively long period (Palena *et al*., 2007), Hamilton and colleagues were able to demonstrate that the poor killing observed was due in great part to a defect in caspase‐dependent apoptotic death in the cells, despite immune antigenicity (Hamilton *et al*., 2014).

Interestingly, in other cellular contexts, such as NSCLC cells, defects in antigen‐presenting machinery associated with immunoproteasome deficiency were found to be a common event in cancers with a more Mes profile and could affect T cell‐mediated cytotoxicity (Tripathi *et al*., 2016).

## 7 Alterations of cell–cell interactions and immunological synapses

**Figure 2:**
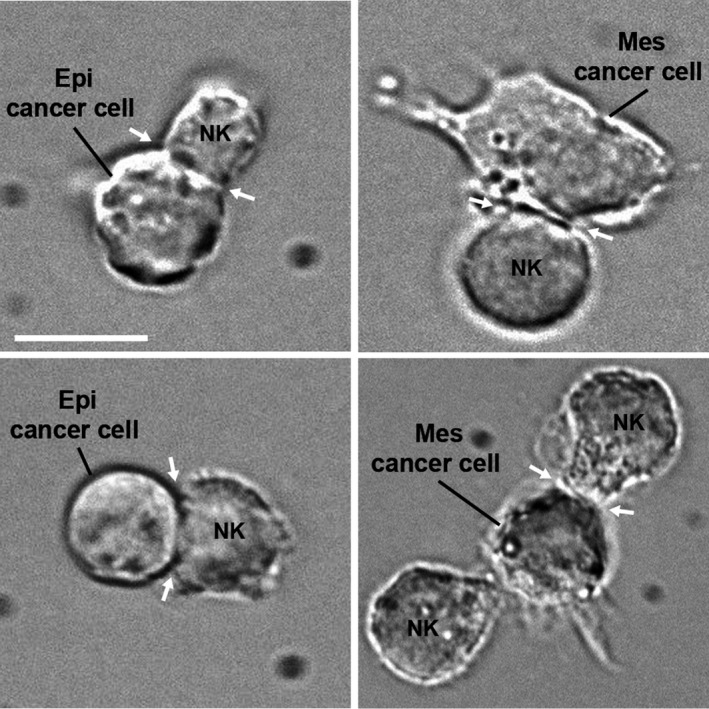
Phase‐contrast microscopy showing conjugates of Epi cancer cell, or Mes cancer cells, with NK lymphocytes (NK92) formed after coculturing the cells for 30 min. Immunological synapses (arrows) are indicated. Scale bar, 10 μm.

Immune killer cells, such as CTLs and NK cells, highly rely on their physical contacts with APCs or target cancer cells for their activity, maturation, production of IFN‐γ and TNF‐α, and lytic functions (Dustin and Long, 2010). These interactions may occur in a form of an ‘immunological synapse’ (IS) (Dustin, 2014; Dustin *et al*., 2010) (Fig. 2). Originally described for T‐cell activation and cytotoxicity, the IS is also critical for NK cell cytotoxicity (Dustin and Long, 2010; Mace *et al*., 2014; Orange, 2008). Formation of the IS in T cells required the coordinated interactions of MHC‐TCR, regulation of cytoskeletal elements such as actin, and the integration of integrin‐based signals and forces in part generated when integrin molecules such as lymphocyte function‐associated antigen‐1 on the T cell, interacted with intercellular adhesion molecule 1 (ICAM‐1) on the target cell. During peptide‐MHC/TCR ligation, integrins undergo conformational changes mediated by phosphorylation cascades, including phosphotyrosine kinase activation, linking integrins to the actin cytoskeleton. The actin cytoskeleton undergoes polymerization at the edge of these active synapses and this further coincides with immune cell flattening and a size increase in the synaptic diameter (Comrie and Burkhardt, 2016). This also leads to the emergence of TCR microclusters coalescing toward the center of the IS into the zone referred to as the central supramolecular activation cluster (Yu *et al*., 2013b). On the other hand, integrin microclusters generally stand in the periphery of the synapse to form a highly contractile zone termed as peripheral supramolecular activation cluster.

Mechanical forces generated at the synapse through intercellular adhesion are also important to regulate adhesion‐based signals and the rearrangement of the actin cytoskeleton. IS involving NK cells seems to obey similar rules except that these cells do not express TCR receptors. Instead, they express activating and inhibitory receptors (DNAM1, NKG2D, 2B4) that may likewise regulate signaling, activity, and dynamic changes in the integrin–actin network at the different stages of NK cell cytotoxicity. Thus again, actin dynamics are tightly controlled at the IS to achieve optimal effector functions. Numerous genetic aberrations have been identified and may alter various stages in NK and CTL cytotoxicity, recognition, F‐actin and microtubule networks, trafficking and docking of lytic granules (not discussed above) (Milner and Holland, 2013; Orange, 2008), thus leading to T cell or NK disorders and severe immunodeficiency. These examples highlight the critical requirement of an operational IS to generate an effective immune response.

Whether some cancer cells act by impeding the IS remains largely unappreciated, although some facts support this hypothesis. For instance, loss of MHC and subsequent MHC/TCR complexes will inevitably hinder much of the activity of the IS involving T cells, as well as its architecture. In line with this, we denoted an alteration of the IS signaling in CTL clones cocultured with Mes MCF7 derivatives, concurrent with reduced expression of MHC class I by the latter, when compared to Epi parental MCF7 cells (Akalay *et al*., 2013). In this line, Dongre *et al*. (2017) recently observed a drop in MHC class I expression in Mes breast carcinoma cells deriving from MMTV‐PyMT mice compared to their more Epi counterparts.

To note, Mes carcinoma cells often show loss of cell polarity, with striking differences in their cytoskeletal organization and content compared to more Epi carcinoma cells (Schliekelman *et al*., 2015). Although we possess only incomplete understanding of the mechanisms involved, we hypothesize that carcinoma cells with a more Mes differentiation could be refractory to establishing mature IS when interacting with CTL. As the establishment of the IS, and activation cascades, relies in part on heterophilic interactions between integrins from the immune effector and ICAM‐1 on target cells, it is easy to foresee how loss of ICAM1 will impede IS formation (Anikeeva *et al*., 2005; Hamai *et al*., 2008). Moreover, changes in the mechanical forces coupled to the actin network could contribute to affect the integrity of the IS and the lytic commitment. Thus, it is likely that perturbation of the actin network in certain cancer cells will make them either more susceptible or more resistant to lymphocyte‐mediated lysis. It was previously observed that strong morphological changes in NSCLC IGR‐Heu cells, associated with alterations of the actin cytoskeleton, promoted resistance to CTL killing (Abouzahr *et al*., 2006). This appeared to occur despite the formation of apparently normal conjugates. At the other end, the recent report by Jachetti and colleagues is relevant in showing how cancer cells can perturb the actin network of effector T cells. By investigating a model of mouse cancer stem‐like cells (CSCs), the investigators elegantly showed that abnormal production of extracellular matrix protein tenascin‐C by CSCs, through interactions with integrin α5β1 at the surface of T cells and interfering with reorganization of the actin cytoskeleton, contributes to inhibition of T‐cell activation (with consequences on their proliferation, and cytokine production). Such observations still need to be fully addressed in the context of Mes carcinoma cells. Additionally, not only are these mechanisms pertinent for T cells and CTL‐mediated killing, but in some circumstances, they may also be pertinent in altering significantly cell‐mediated lysis by NK or other killer cells.

## 8 Immune resistance to NK cells

Natural killer cells are effector lymphocytes of the innate immune system controlling tumor growth during cancer initiation and progression (Vivier *et al*., 2008). Although the contribution of NK cells during the different steps of tumor progression is still unclear, presumably varying between histological and cancer types (Gentles *et al*., 2015), their behavior with Mes carcinoma cells remains to be fully addressed. Relevant for this discussion is the observation that NK cells in cancer tissues are rarely found in direct contact with the tumor mass, but rather present in the stroma (Halama *et al*., 2011). Other studies showed that NK cells can infiltrate tumors, but in this case, NK cells may have impaired capabilities to kill cancer cells (Mamessier *et al*., 2011; Platonova *et al*., 2011). In this context, one issue to consider is that cancer cells undergoing EMT, in association with their increased capacity to invade the stroma, and propensity to become circulating tumor cells (CTCs), are hardly detectable in the stromal compartment or in the bloodstream, due to common features with fibroblastic cells and lack of optimized approaches to detect them.

To note, reduction of MHC class I by certain cancer cells can be perceived as a ‘missing self’ by the inhibitory receptors KIRs of NK cells that usually interact with MHC class I (Vivier *et al*., 2008). As a result, this could render cancer cells susceptible to NK cells. However, because NK cell activation and killing is regulated by a combination of activating and inhibiting receptors and their related ligands (Chester *et al*., 2015), reduction or loss of MHC class I is rarely sufficient to trigger NK cell‐mediated lysis. Cancer cells may again exploit various strategies to compensate this loss such as upregulation of the nonclassical MHC class I proteins HLA‐G (Carosella *et al*., 2015) and HLA‐E (Braud *et al*., 1998), able to restrain NK activity through KIRs and CD94/NKG2A, respectively. There is also evidence in some cancer cells for an increase in resistance to perforin/granzyme B‐mediated cell death by immune cells (Ben Safta *et al*., 2015), reduction in cell surface MHC class I‐related chain (MIC) proteins, MICA and MICB, and UL16 binding proteins, ligands for NK‐activating receptor‐activating NKG2D (Malladi *et al*., 2016). Furthermore, the production by certain cancer cells of soluble forms of MICs could act as a decoy for NK cells while promoting degradation of NKG2D (Groh *et al*., 2002; Raffaghello *et al*., 2004).

## 9 EMT‐mediated immune resistance to NK cells

In a recent report, we observed that NSCLC tumor subclones with Mes features exhibited an increased propensity to resist NK cell‐mediated lysis compared to more Epi subclones. This immunoresistant phenotype was at least partly due to defective immune synapse signaling (Terry *et al*., 2017). Further investigations are needed to unravel the detailed mechanisms as to why those carcinoma cells are more prone to inactivate the IS when interacting with NK cells. Aside from our observation, one significant breakthrough came from Hamilton *et al*. who demonstrated that high levels of the EMT‐related factor Brachyury reduced the susceptibility of carcinoma cells not only to CTLs, but also to NK cells, lymphokine‐activated killer, FAS, and TRAIL‐induced cell death. In a recent report investigating the lung cancer setting, the same group further found a potential role for estrogen receptor α in promoting both Mes characteristics and resistance to immune‐mediated cytotoxicity (Hamilton *et al*., 2016). By contrast, the study of López‐Soto *et al*. (2013) described how EMT in colon cancer cells, following SNAIL overexpression, could instead render cancer cells more sensitive to NK‐mediated lysis in a manner that appears dependent on NKG2D ligands upregulated in the cells undergoing EMT. These investigators obtained similar results in immortalized keratinocytes stimulated by TGF‐β.

## 10 EMT, immune checkpoints, and immunosuppression

Similar to MDSC or Treg cells present in the TME, cancer cells can express immunoregulatory enzymes, immunosuppressive cytokines, or immune checkpoint ligands to modulate efficacy of the immune response and its duration. Among those, TGF‐β has been one of the most studied so far (Massague, 2008). It is a known EMT driver whose expression is often upregulated in Mes cancer cells. Moreover, TGF‐β can impair maturation, differentiation, or activation of both innate and adaptive immune cells, including NK cells, DCs, macrophages, neutrophils, and CD4^+^ and CD8^+^ T cells reviewed in Tu *et al*. (2014). TGF‐β can inhibit the cytotoxic T‐cell functions by altering the expression of cytotoxic gene products including perforin, granzyme A, granzyme B, Fas ligand, as well as IFN‐γ (Joffroy *et al*., 2010; Thomas and Massague, 2005). In addition, it can inhibit their differentiation into central memory T cells (Takai *et al*., 2013). In prostate cancer cell lines, TGF‐β led to MHC class I downregulation (Chen *et al*., 2015). TGF‐β secreted by cancer cells was also documented to induce Treg cells from CD4^+^ T cells, based on induction of Foxp3 expression in cocultures (Joffroy *et al*., 2010). In NK cells, TGF‐β impaired the cytokine production (Wilson *et al*., 2011) and their cytolytic activity at least by downregulating expression of NKG2D (Lee *et al*., 2004).

Clinical success of novel immunotherapies targeting immune checkpoints also underlines the importance of immune checkpoint ligands in the control of the immune response (Burstein *et al*., 2017). Expression of checkpoint molecules such as PD‐L1 dampens the immune response and can hold T cells from killing target cancer cells. Indeed, PD‐L1 is a ligand for the cell surface receptor PD‐1 especially expressed by T lymphocytes which upon signaling (Crespo *et al*., 2013; Zou *et al*., 2016) will reduce T‐cell activity, and in the case of CTLs, inhibit T‐cell cytotoxic activity, eventually enforcing an exhaustion state (van der Burg *et al*., 2016; Zou *et al*., 2016). When antibodies block these proteins, T cells may at least partially restore their activity. PD‐L1 expression can be naturally induced by inflammatory cytokines including IFN‐γ and TNF‐α, which limits immune responses upon inflammatory conditions. In cancer, various signals and alterations can drive PD‐L1 expression as an immunosuppression mechanism (Chen *et al*., 2016a; O'Donnell *et al*., 2016). Interestingly, both *TGF*β*1* and *CD274* (encoding for PD‐L1) genes can be induced under hypoxic conditions, either directly via hypoxia‐induced factors (HIFs) or indirectly through related factors (Barsoum *et al*., 2014; Hasmim *et al*., 2013; Noman *et al*., 2014).

Chen *et al*. (2014) have identified a molecular link between EMT and more abundant expression of PD‐L1 in human lung tumors. *In vitro* and *in vivo* experiments demonstrated that downregulation of miR‐200s and ZEB1 overexpression not only drive EMT but also may lead to upregulation of PD‐L1. Beyond showing the regulation of PD‐L1 by the ZEB1/miR‐200 axis, perhaps one of the most intriguing observations was the association of these events with exhaustion of intratumoral CD8^+^ T lymphocytes, which ultimately promoted the development of metastases in mice. Further work by this group also indicated a role for bone morphogenetic protein‐4 (BMP4) to regulate PD‐L1 expression (Chen *et al*., 2016b). As a follow‐up question, it would be interesting to see whether additional immune checkpoints, other than PD‐L1, likewise associated with a more Mes tumor phenotype (Mak *et al*., 2016), play a role in T‐lymphocyte exhaustion.

These studies are also important because it is expected that tumors with high PD‐L1 expression, or other inhibitory checkpoints ligands, will be the best protected against the endogenous immune response, while patients with such tumor characteristics are also believed to be good candidates for immunotherapy, with better efficacy of immune checkpoint blockade, and reinvigoration of the immune response. Consistent with this notion, high expression of PD‐L1 in metastatic NSCLC patients was able to predict, at least to some degrees, response to anti‐PD1 treatment (Garon *et al*., 2015). This led to the development of clinical studies using predefined PD‐L1 expression level into the inclusion criteria for patients. Recent approval of pembrolizumab in the first‐line metastatic NSCLC has been established under such conditions (Reck *et al*., 2016). Nevertheless, PD‐L1 alone might not be sufficient as a predictive marker of response to anti‐PDL1 monoclonal antibodies (Topalian *et al*., 2016). Promising markers of response are currently under evaluation including the mutational load (Hugo *et al*., 2016; McGranahan *et al*., 2016; Rizvi *et al*., 2015; Rosenberg *et al*., 2016; Snyder *et al*., 2014); the MSI (Le *et al*., 2015), somatic copy number alterations (Davoli *et al*., 2017), or immune‐related signatures (Prat *et al*., 2017). The presence of tumor‐resident CD103^+^ TILs could also represent a good candidate marker of response to immunotherapy as this TIL population seems to express PD‐1 (Djenidi *et al*., 2015; Komdeur *et al*., 2016).

We should consider that optimal response would likely depend on the global expression pattern of the immune checkpoint receptors and the combined expressions of their ligands. In addition to PD‐L1, ligands expressed at the surface of cancer cells and immunosuppressive cells include PD‐L2, galectin‐9, CEACAM1, CD70, CD137L, OX40L, ICOSL, and corresponding receptors: T‐cell immunoglobulin mucin receptor 3 (TIM3; *HAVCR2*), lymphocyte activation gene 3 (LAG3) regarding the coinhibitory receptors, and costimulatory receptors CD27, CD137, OX40, GITRL, or ICOS (Mahoney *et al*., 2015; Melero *et al*., 2015).

Current studies aimed at unraveling how EMT may promote immunosuppression and to expand the list of the TME cellular components involved (Fig. 1). Kudo‐Saito *et al*. (2009) showed that forced expression of Snail in mouse melanoma B16F10 cells enhanced the EMT program in these cells, in conjunction with increased thrombospondin 1 production, induced an impairment of DC maturation while allowing expansion of a population of suppressive Treg‐like CD4^+^ Foxp3^+^ cells in cell cultures. Similarly, in syngeneic mice, tumors formed by mock‐transfected B16F10 cells revealed infiltrating CD8^+^ T cells, whereas tumors derived from Snail‐transduced B16F10 showed less tumor‐infiltrating CD8^+^ T cells, more Treg, and more metastasis to the lungs. Subsequent work from this group also pointed out the critical role of the chemokine CCL2 in mediating the described immunosuppressive effects, with possible effects on recruitment of other immunosuppressive CCR2^+^ populations such as MDSC and macrophages (Kudo‐Saito *et al*., 2013). Others found in the MMTV‐PyMT mouse model of breast cancer that tumors arising from Mes carcinoma cell lines, or Snail^high^ cell populations, exhibited more Tregs and protumoral macrophages compared to their more epithelial, or snail^Low^, counterparts (Dongre *et al*., 2017). Using various cancer models, Ricciardi and colleagues observed that exposure to inflammatory cytokines can endow cancer cells undergoing EMT with a number of immunomodulatory effects depending on the cancer cell origin. This included interference with proliferation, differentiation, and apoptosis of NK, T‐, and B‐cell populations (Ricciardi *et al*., 2015). They underlined a role of the indoleamine‐2,3‐dioxygenase (IDO) pathway to explain the deleterious effects observed on T cells after inflammatory‐induced EMT (Platten *et al*., 2012; Ricciardi *et al*., 2015). Yu *et al*. reported evidence suggesting that hypoxia‐induced EMT in hepatocellular carcinoma (HCC) induces an immunosuppressive TME to promote metastasis (Ye *et al*., 2016). On a mechanistic standpoint, they showed that hypoxia‐induced EMT in HCC cells promoted increased expression of CCL20 in a HIF‐1α dependent manner, which through its action on monocyte‐derived macrophages, and expression of IDO by these cells, led to decreased proliferation of CD4^+^ and CD8^+^ T cells, while promoting the expansion of immunosuppressive Treg cells.

Collectively, these studies provide important insights into the diversity of actors and routes exploited by Mes cancer cells to escape the immune system. Moreover, this highlights the need to gain further knowledge on the composition of TME niches in various tumor sites and how those establish.

## 11 Cancer EMT signatures and immune activation

**Figure 3:**
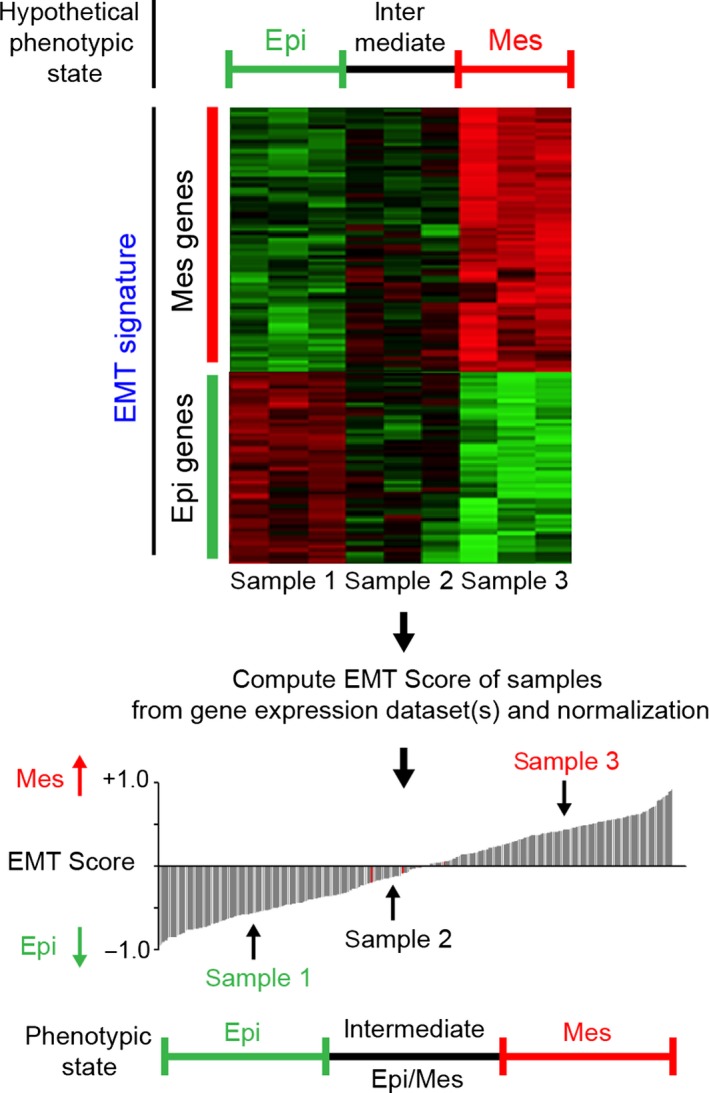
Schematic diagram illustrating the potential utility of EMT scoring. An EMT score is inferred after interrogating a gene expression dataset for previously published or in‐house‐generated EMT signatures. Cell line or tumor samples can be compared and sorted from most Epi to most Mes based on their EMT score. An arbitrary threshold may be used to define Epi, intermediate Epi/Mes, or more advanced Mes states.

EMT scoring based on cancer‐specific transcriptomic signatures was pioneered by various groups (Byers *et al*., 2013; Mak *et al*., 2016; Tan *et al*., 2014). This EMT score was developed based on expression profiling of several hundred genes (Tan *et al*., 2014), and this score has been used to assess (semiquantitatively) the position of cancer cells in the EMT spectrum (Fig. 3). Most tumor types and corresponding cell lines exhibit a wide range of EMT scores, with values ranging from −0.8 for most Epi to +0.6 for most Mes features. While the majority of colon carcinomas and corresponding cell lines appear more Epi on average, some tumors do exhibit Mes‐like phenotypes likely corresponding to the newly established Mes molecular subtype (Punt *et al*., 2017). Other tumor types such as breast, ovarian, pancreatic, lung, and prostate tumors and cell lines have an average value of 0 but show a large spectrum of values. Remarkably, renal carcinomas display strong Mes features, perhaps because they derive from an epithelium initially originating from the metanephric mesodermal mesenchyme. On the other hand, melanomas are expectedly quite Mes but not as mesenchymal as osteosarcoma. The EMT score reflects, in part, the EMT status of the cancer cells but also contributions from the stromal fibroblasts in the tumor sample. Indeed, transcriptomes of breast tumors depleted from their stroma by microdissection show that luminal and ErbB2 subtypes are Epi‐like, whereas basal‐like and claudin‐low tumors have pronounced EMT scores. Generally, the EMT spectrum of cell lines spans the same spectrum as their corresponding tumor types, suggesting that cell lines maintain the EMT features from their tumors of origin (Tan *et al*., 2014).

Among molecular alterations associated with EMT, pathway analysis revealed association between high EMT score and high expression of several immune checkpoints including PD1, PD‐L1, PD‐L2, B7‐H3, OX40, OX40L, CD137, TIM3, LAG3, and CTLA4 (Mak *et al*., 2016). Lou *et al*. (2016) confirmed these observations in three independent datasets of lung cancer patients with early or advanced NSCLC adenocarcinomas. Additional reports suggested an association between PD‐L1 expression and EMT in NSCLC and HNSCC (Kim *et al*., 2016; Ock *et al*., 2016b). In their study, Lou *et al*. further found that tumors harboring high Mes content were associated with increased infiltration by TILs and Treg cells, and increased expression of costimulatory molecules (CD80, CD86). Furthermore, they showed elevated endogenous immune activation in these tumors, as revealed by a higher expression of IFN‐γ, IFN‐γ‐inducible CXCL10 and IDO, among many other upregulated genes (Lou *et al*., 2016). In the same study, the B7 family of immunomodulatory molecule, B7‐H3 (CD276), was correlated with the EMT score and found to have a prognostic value for disease‐free survival and overall survival. By contrast, neither PD‐L1, other immune checkpoints, nor EMT were found to have such prognostic value. No data were available on whether some of these patients received immunotherapy regimens during their treatment history. Nevertheless, these studies highlight the rationale to test gene expression‐based EMT signatures as a predictive marker to select patients for anticheckpoint therapies. Importantly, the investigators demonstrated no association between EMT and mutational burden, which raises the interesting possibility that evaluation of both mutational load and EMT status could help improve patient selection (Lou *et al*., 2016).

By investigating the Epi marker ESRP1 into The Cancer Genome Atlas dataset, Yao *et al*. (2016) identified a link between enhanced Mes features in ESRP1‐low melanoma and high infiltrating lymphocyte activity, as assessed by a two‐gene signature (*PRF1* encoding for perforin and *GZMA* encoding for granzyme A). The ESRP1‐low/Mes high group further showed better overall survival compared to groups expressing full‐length or truncated forms of ESRP1. The authors suggested this subgroup of melanoma patients as well suited for immunotherapy intervention. In this regard, in the recent study of Hugo *et al*. (2016) analyzing biopsies of anti‐PD1‐treated advanced melanoma patients, EMT signatures and Mes‐related genes were found to be upregulated among nonresponding relative to responding patients, thus suggesting that the Mes features of the primary tumor may be associated with innate anti‐PD‐1 resistance. However, T cell‐related genes such as CD8A/B, PD‐L1, LAG3 (T‐cell checkpoint genes), and IFN‐γ did not appear differentially expressed in nonresponding compared to responsive tumors.

In bladder cancers, patients with tumors characterized by an Epi (luminal) phenotype had a better response rate while treated with anti‐PD‐L1 therapy compared to those harboring basal subtype (Rosenberg *et al*., 2016) expressing numerous Mes markers (Choi *et al*., 2014; Kamat *et al*., 2016). Reports also suggest that, similar to what has been found in the claudin‐low subtype of breast cancer, there exists a claudin‐low subtype of high‐grade muscle‐invasive bladder cancer, defined by highly apparent Mes features. Moreover, despite being infiltrated by immune cells, this subtype was actively immunosuppressed (Kardos *et al*., 2016).

In considering EMT signatures, it is important to remember that a rich stromal contingent present within the tumor specimen can contribute to disturbing the assessment of the cancer EMT score. In particular, it remains extremely complicated in this type of analysis to clearly distinguish Mes cancer cells from CAFs. EMT‐TFs are expressed by Mes cells in the normal and tumor stroma; moreover, they may contribute to the emergence of CAF populations, providing a specialized microenvironment also with a potential role in resistance to treatment and immune response. To our knowledge, the published data regarding association between EMT and immune signatures have not totally addressed the potential contribution of cancer *vs* stroma in Mes features. Interestingly, in colon cancer, where the stromal fraction should account for most of the Mes contents, Becht *et al*. (2016a), using transcriptomics, were able to identify an immune‐related stromal/Mes subtype, characterized by an immunosuppressive signature correlating with a high density of fibroblasts likely producing chemokines, cytokines, inflammatory, and angiogenic factors.

Altogether, these reports indicate that there is some need for caution when considering EMT scoring as a predictor of clinical response for immunotherapy. More research and prospective studies are urgently needed to test this hypothesis across numerous cancer types (and disease subtypes).

## 12 Targeting EMT to attenuate immunoresistance and improve susceptibility to cytotoxic antitumor immune response

In our previous work, we observed that inhibition of TGF‐β signaling can increase susceptibility of Mes carcinoma cells to CTL and NK cell‐mediated lysis (Akalay *et al*., 2015; Terry *et al*., 2017). In theory, such a strategy could be compatible with immunotherapy to facilitate response at multiple levels. Notably, Mes carcinoma cells often display elevated TGF‐β signaling contributing to maintain both cell plasticity and immune resistance of these cells. TGF‐β is likewise a well‐known immunosuppressive substance. Of therapeutic relevance, multiple molecules targeting TGF‐β signaling are currently tested in clinical studies (Chretien *et al*., 2014; de Gramont *et al*., 2017). We envision that Mes carcinoma may be avid targets for such compounds. Because phenotypic and functional EMT‐driven cancer cell plasticity allows malignant cells to adapt to selective pressures including the immune response, and leads to the development of resistance to targeted therapies, targeting EMT‐driven cancer cell plasticity may represent an innovative approach to cancer therapy. Given the dynamic nature of the EMT‐driven epithelial plasticity, targeting EMT may request to concurrently target several processes in order to be successful. Agents that can be used to target the EMT may include extracellular inducers of EMT, the transcription factors that orchestrate the EMT transcriptome, the downstream effectors of EMT, and the canonical pathways known to be involved in EMT, such as the signal transduction pathways driven by activation of TGF‐βR, EGFR, and AXL, as well as emerging pathways such as epigenetic therapies, glycosylation pathways, or EMT‐associated metabolic alterations (Davis *et al*., 2014; Malek *et al*., 2017). Recent studies from high‐throughput analyses have now selected a number of candidate compounds that could preferentially alter the viability of carcinoma cells with high Mes features (Byers *et al*., 2013; Chua *et al*., 2012; Ock *et al*., 2016a; Tan *et al*., 2014). T cells specifically educated or engineered to target Mes cancer cell populations, or vaccines designed to target those, could also represent interesting strategies to control the expansion of Mes cancer cell populations. Previous studies demonstrated some efficacy using vaccines directed to the EMT drivers Brachyury, TWIST1, or CRIPTO (Ardiani *et al*., 2014; Hamilton *et al*., 2013; Ligtenberg *et al*., 2016; Palena *et al*., 2007). More experimental studies are now needed to evaluate the clinical efficacy in monotherapy and in combination with approved immune checkpoint blockers.

Recent reports suggested that two widely used compounds, the EGFR inhibitor erlotinib, and fulvestrant, an antagonist of ER, may be efficient at decreasing Mes features of lung cancer cells as well as for improving their lysis by the immune effector cells (Dominguez *et al*., 2016; Hamilton *et al*., 2016). In addition, as suggested in our previous studies, it could be interesting to target autophagy when Mes carcinoma cells exhibit an autophagic state (Akalay *et al*., 2013).

Aside from the context of EMT, other compounds have been tested that should be considered to enhance susceptibility to immune killer cells (Bommarito *et al*., 2016; He *et al*., 2013). Agents inhibiting the expression of PD‐L1 through blockade of the AKT/PI3K/MTOR or the RAS/MEK signaling pathways, and more generally drivers of PD‐L1 expression, could help sensitize cancer cells to immunotherapy at the onset in various tumor types (Alsuliman *et al*., 2015; Lastwika *et al*., 2016; Loi *et al*., 2016; Peng *et al*., 2016). Of note, immune cells could be pharmacologically manipulated. Elegant work by Parameswaran *et al*. (2016) showed how pharmacological GSK3 inactivation in NK cells can restore NK cell cytotoxicity in patients with AML.

Although all these approaches could be efficient, heterogeneity of tumors, the diversity of these cancer cell populations, their high plasticity, and adaptability to stress will remain a major issue. Therefore, to achieve a durable response, it will be essential to develop strategies able to limit the tumor heterogeneity and cell plasticity. We presume that novel strategies targeting both tumor contingent and microenvironmental context might improve efficacy of cancer treatments. In this case, hypoxia could be an attractive target to limit tumor heterogeneity, cell plasticity, the expansion of immunosuppressive cells, and secretion of immunosuppressive substances, while rendering cancer cells more susceptible to immune attacks (Noman *et al*., 2015; Paolicchi *et al*., 2016; Wilson and Hay, 2011). One of the main caveats will be to design potent and specific inhibitors of HIFs. Alternatively, drugs affecting developmental pathways and plasticity of cells must be considered (Malladi *et al*., 2016; Pattabiraman and Weinberg, 2014). Similarly, epigenetic modifiers such as demethylating agents, or histone deacetylase inhibitors, could be exploited for blocking this plasticity or as priming agents to boost the efficacy of immunotherapy (Chretien *et al*., 2014; Saleh *et al*., 2016).

## 13 Surrogate biomarkers of EMT‐driven cancer cell plasticity and the immune microenvironment

The recent success of immunotherapies provides some evidence that additional approaches to genomic‐driven precision medicine may be rewarding. Indeed, although recent progress in the characterization of genetic abnormalities has resulted in the multiplication of clinical trials driven by genomic alterations (e.g., ProfiLER, SHIVA, MOSCATO, and MD Anderson genomic‐driven clinical trials), an important limitation of this approach is the relatively low average (30–40%) of targetable genomic alterations, leading to poor recruitment rate in early‐phase clinical trials, and potentially, a limited number of patients who may benefit from the targeted therapy (Barlesi *et al*., 2016; Massard *et al*., 2017). Concurrently, a platform that will allow screening real‐life biospecimens for surrogates of EMT‐driven cancer cell plasticity, to foster inclusions in clinical trials evaluating drugs targeting EMT and the immune microenvironment, is urgently needed. Of note, the immune microenvironment including the degree of activation of specific pathways and immune subpopulations is the subject of active bioinformatic research (Becht *et al*., 2016b; Galon *et al*., 2013; Gentles *et al*., 2015). However, this approach has some limitations: (i) the unknown relevance of the gene signature for the study of EMT in advanced stages of the disease; (ii) technological challenge to generate gene expression profiles from formalin‐fixed paraffin‐embedded (FFPE) samples; (iii) the inability to distinguish the contribution of stroma when no selection of the region of interest is performed; (iv) transcriptional EMT signatures identify phenotypic changes, but it is now recognized that overexpression of EMT‐TFs without phenotypic changes may be associated with functional plasticity; (v) the challenge to evaluate the dynamics of gene expression changes in serial tissue biopsies. In order to address these challenges, an effort to characterize metastatic disease, the development of technologies dedicated to FFPE samples (NanoString and HTG EdgeSeq technologies), and the identification of new surrogate biomarkers of EMT‐driven cancer cell plasticity in carcinoma cells that are not expressed by stroma cells are needed.

Finally, because EMT is reversible, it is critical to consider the dynamics of this process. While it is difficult to perform serial tumor biopsies, the use of liquid biopsies for a noninvasive monitoring of disease evolution is increasingly implemented (Haber and Velculescu, 2014; McGranahan and Swanton, 2017). CTCs consist of cancer cells with very different EMT phenotypes along the EMT spectrum including transitional/hybrid states between Epi and Mes (Kang and Pantel, 2013; Vona *et al*., 2000). Methods that rely on the display of cell surface Epi markers such as EpCAM by CTCs may well miss capturing a sizeable, clinically relevant portion of the CTCs (Farace *et al*., 2011). ISET is an antibody‐independent whole blood filtration‐based approach for CTC isolation that relies on the larger size of all types of circulating rare cells compared to most leukocytes (Laget *et al*., 2017). Feasibility of immunostaining for vimentin, pan‐cytokeratin, EpCAM, EGFR, Ki67, and c‐MET has been previously reported (Krebs *et al*., 2012; Laget *et al*., 2017; Lecharpentier *et al*., 2011). EMT can potentially occur in blood or lymph vessels; however, given that the half‐life of single CTC is very short (Meng *et al*., 2004), it is unlikely that these cells can be induced to undergo EMT. Nonetheless, they could do so beforehand, or once aggregated into microemboli which can reside for much longer in capillaries in close contact with platelets that release TGF‐β (Labelle *et al*., 2011). Much work remains to be conducted to adequately assess the role of EMT in CTCs and whether they can mark refractoriness to targeted therapeutics and immunotherapies (Alix‐Panabieres *et al*., 2017). Longitudinal studies evaluating changes over time of EMT‐TFs expression and other surrogates of EMT‐driven cancer cell plasticity may be informative, but are lacking information on the immune microenvironment. Immunomonitoring in blood may provide additional information compared to the *in situ* immune microenvironment, although this remains to be shown.

## 14 Conclusion

Recent advances in the field of cancer immunotherapy have revolutionized the management of patients with melanoma, NSCLC, renal cell carcinomas, bladder carcinomas, HNSCC, ovarian carcinomas, and lymphomas (Burstein *et al*., 2017). We are still at the beginning of an exciting period of discovery and improvement of these therapies. One of the biggest challenges toward such improvement is to better understand the mechanisms at play in the naturally acquired resistance seen in some patients, as well as in therapy‐induced resistance seen in subgroups of patients, on or after treatment, who do initially respond to immunotherapy. Additionally, it will be critical in this effort to identify potential targets responsible for this resistance and develop new strategies able to eliminate the cancer cell‐resistant clones or prevent their emergence. The link between EMT and immune recognition and killing of cancer cells is now well established. Numerous observations now provide relevant clues to how Mes carcinoma cells could contribute such resistance, while pointing those as promising targets to consider for improving immunotherapy regimens and develop predictive markers of response. In this perspective, we reason that epithelial‐mesenchymal plasticity, a critical program for carcinoma progression and metastasis, is a central driver of not only tumor malignancy but also immune regulation and antitumor response shaping. Clearly, EMT is a key process that is critical for immune resistance but also a potent driver for the activation of an immunosuppressive network within the TME. Therefore, targeting EMT may offer important perspectives for the current immunotherapy approaches for advanced tumors.
